# Supplementary figures and images for: Comparative Transcriptomics Reveals Genes Commonly Induced by Distinct Stressors in Chlamydia
Source: bioRxiv. 2025 Dec 30:2025.12.30.696969. Preprint. [Version 1] doi: 10.64898/2025.12.30.696969 (PMC12776308; doi:10.64898/2025.12.30.696969)

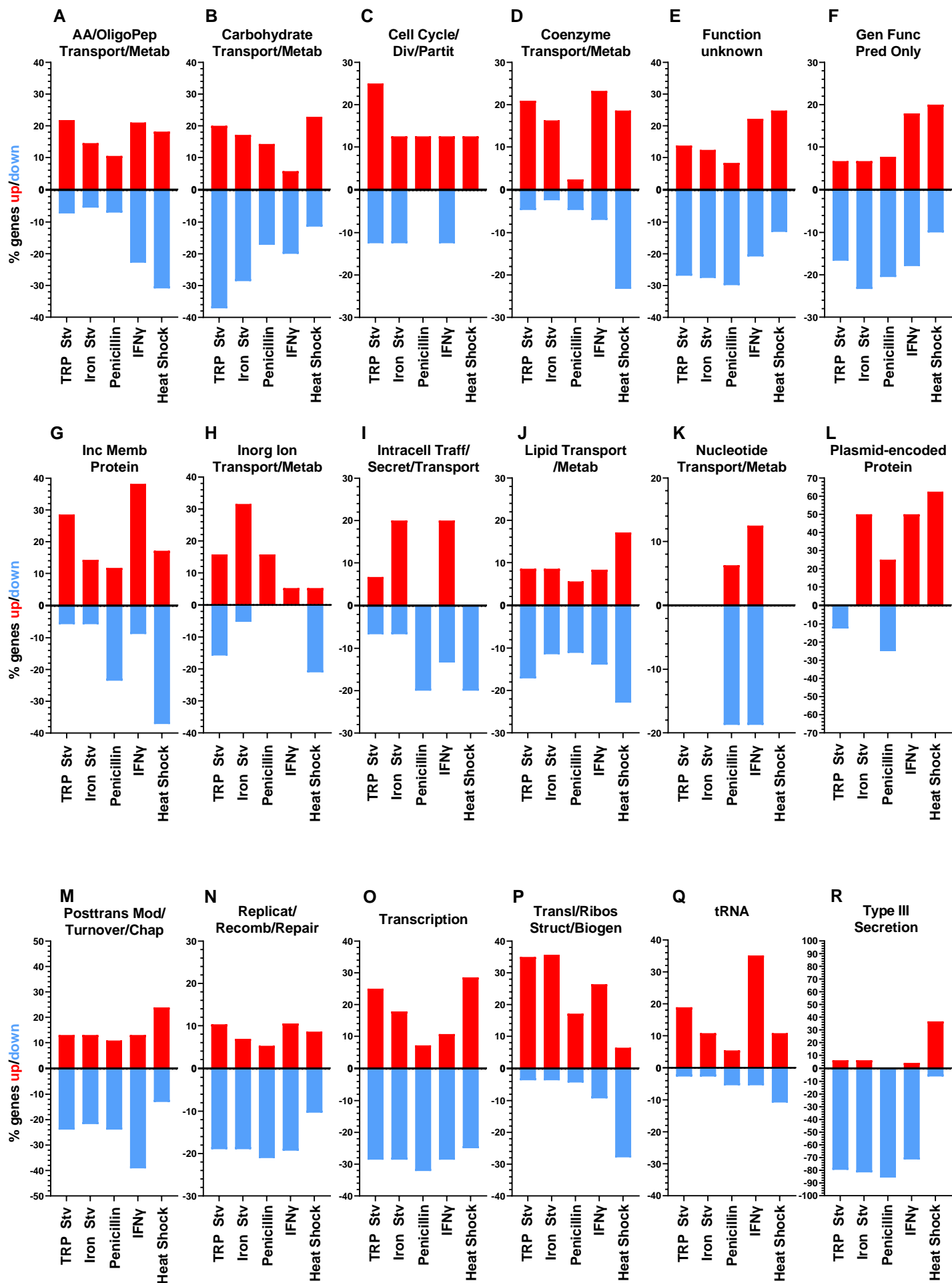

Supplement: Supplement 2 [file media-2.pdf]
